# Erythropoietin attenuates motor neuron programmed cell death in a burn animal model

**DOI:** 10.1371/journal.pone.0190039

**Published:** 2018-01-31

**Authors:** Sheng-Hua Wu, I-Cheng Lu, Su-Shin Lee, Aij-Lie Kwan, Chee-Yin Chai, Shu-Hung Huang

**Affiliations:** 1 Department of Anesthesiology, School of Medicine, College of Medicine, Kaohsiung Medical University, Kaohsiung, Taiwan; 2 Department of Anesthesiology, Kaohsiung Medical University Hospital, Kaohsiung Medical University, Kaohsiung, Taiwan; 3 Department of Anesthesiology, Kaohsiung Municipal Hsiao-Kang Hospital, Kaohsiung Medical University, Kaohsiung, Taiwan; 4 Department of Surgery, School of Medicine, College of Medicine, Kaohsiung Medical University, Kaohsiung, Taiwan; 5 Division of Plastic Surgery, Department of Surgery, Kaohsiung Medical University Hospital, Kaohsiung Medical University, Kaohsiung, Taiwan; 6 Orthopaedic Research Center, Kaohsiung Medical University, Kaohsiung, Taiwan; 7 Center for Stem Cell Research, Kaohsiung Medical University, Kaohsiung, Taiwan; 8 Division of Neurosurgery, Department of Surgery, Kaohsiung Medical University Hospital, Kaohsiung Medical University, Kaohsiung, Taiwan; 9 Department of Pathology, Kaohsiung Medical University Hospital, Kaohsiung Medical University, Kaohsiung, Taiwan; 10 Department of Pathology, School of Medicine, College of Medicine, Kaohsiung Medical University, Kaohsiung, Taiwan; Institute of Biochemistry and Biotechnology, TAIWAN

## Abstract

Burn-induced neuromuscular dysfunction may contribute to long-term morbidity; therefore, it is imperative to develop novel treatments. The present study investigated whether erythropoietin (EPO) administration attenuates burn-induced motor neuron apoptosis and neuroinflammatory response. To validate our hypothesis, a third-degree hind paw burn rat model was developed by bringing the paw into contact with a metal surface at 75°C for 10 s. A total of 24 male Sprague–Dawley rats were randomly assigned to four groups: Group A, sham-control; Group B, burn-induced; Group C, burn + single EPO dose (5000 IU/kg i.p. at D0); and Group D, burn + daily EPO dosage (3000 IU/kg/day i.p. at D0–D6). Two treatment regimens were used to evaluate single versus multiple doses treatment effects. Before sacrifice, blood samples were collected for hematological parameter examination. The histological analyses of microglia activation, iNOS, and COX-2 in the spinal cord ventral horn were performed at week 1 post-burn. In addition, we examined autophagy changes by biomarkers of LC3B and ATG5. The expression of BCL-2, BAX, cleaved caspase-3, phospho-AKT, and mTOR was assessed simultaneously through Western blotting. EPO administration after burn injury attenuated neuroinflammation through various mechanisms, including the reduction of microglia activity as well as iNOS and COX-2 expression in the spinal cord ventral horn. In addition, the expression of phospho-AKT, mTOR and apoptotic indicators, such as BAX, BCL-2, and cleaved caspase-3, was modulated. Furthermore, the activity of burn-induced autophagy in the spinal cord ventral horn characterized by the expression of autophagic biomarkers, LC3B and ATG5, was reduced after EPO administration. The present results indicate that EPO inhibits the AKT-mTOR pathway to attenuate burn-induced motor neuron programmed cell death and microglia activation. EPO can modulate neuroinflammation and programmed cell death and may be a therapeutic candidate for neuroprotection.

## Introduction

Despite recent improvements in burn injury outcomes, post-burn morbidity is high and remains a challenge for clinicians. Patients who experience major burns are at a risk of various adverse outcomes for many years after the initial injury, including altered metabolism in most body tissues, nervous system-related morbidity, and musculoskeletal complications [1–4]. Aggressive treatment after burn injury, such as nutrient support and rehabilitation, provides only partial recovery with residual defects [5–7]. Skeletal muscle wastage after burn injury may also contribute to long-term morbidity; therefore, the understanding of underlying molecular mechanisms is crucial for developing novel treatment solutions. Most related studies have focused particularly on cellular and molecular mechanisms in muscle cells. The adverse outcomes for patients with persistent muscle wastage after burn injury are similar to those following any severe trauma, including imbalanced inflammatory responses and muscle cell apoptosis [8–13]. Our previous study revealed that burn-induced neuromuscular dysfunction is associated with motor neuron apoptosis in the spinal cord ventral horn and subsequently causes denervation muscle atrophy [14]. Therefore, additional investigation on the underlying mechanisms and possible targeted protective strategies is warranted.

Erythropoietin (EPO) is a type of erythropoiesis-stimulating agent that can be used for erythropoiesis regulation [15]. Epoetin (recombinant human EPO) has been approved for the treatment of anemia in patients with chronic kidney disease or those receiving chemotherapy for more than two decades. In addition to hematopoietic effects, accumulated evidence has suggested that EPO can be used as a tissue-protective agent and that it plays a role in increasing oligodendeogenesis [16], preventing inflammation [17–19], and reducing apoptosis [20, 21]. EPO acts on its receptor (EPOR) to activate different kinases and intracellular signaling pathways in various nonhematopoietic tissues, such as in the kidney, endothelial cells, central nervous system (CNS), heart, and reproductive tract [22–26]. EPO has been used to protect against neurotoxicity [21], ischemia/reperfusion injury [27, 28], and neurologic diseases [29, 30]. n in *vitro* or *vivo* models, EPO has been effective in improving functional outcomes after the experimental induction of traumatic brain injury [31–33] and spinal cord injury [34], limiting neuronal damage-associated epilepsy [35, 36] and reducing chemotherapy-induced peripheral neurotoxicity [37, 38]. Clinical trials on systemic EPO have also yielded encouraging results [39, 40]. The possible mechanism underlying EPO-mediated neuroprotection is mediated through antiapoptotic responses in neurons, endothelial responses by increasing blood flow and oxygen delivery for increased vascular relaxation and angiogenesis, and anti-inflammation activities [23]. Moreover, EPO is considered a systemic protective cytokine [41].

However, only a few studies have reported on the effectiveness of EPO treatment on muscle wastage and motor neuron apoptosis [42, 43], and the possible underlying mechanisms remain ambiguous. The present study used two different EPO regimens following burn injury to evaluate single versus multiple doses treatment effects in preventing programmed cell death. We further analyzed the anti-neuroinflammation effects of EPO based on microglia activation and inducible nitric oxide synthase (iNOS) and cyclooxygenase-2 (COX-2) expression in the spinal cord ventral horn. The effects of EPO on the phospho-protein kinase B (p-AKT)-mechanistic target of rapamycin (mTOR) signaling pathway were also evaluated in the burn injury model.

## Materials and methods

### Experimental animals

Male Sprague–Dawley (SD) rats (n = 24) weighing 150–175 g were used in this study. The study protocol was approved by the Institutional Animal Care and Use Committee of Kaohsiung Medical University (IACUC Approval Number: 106047). All rats were housed in plastic cages with soft bedding under 12-h light–dark cycles with free access to food and water.

### Experimental design

The rats were randomly divided into four groups containing six animals each, as following:

1. Group A (sham-control, n = 6) rats were subjected to sham burn without receiving drugs and served as untreated controls for all experimental groups,
2. Group B (burn-induced, n = 6) rats were subjected to burn injury and served as the untreated group,
3. Group C (burn + single EPO dose, n = 6) rats were subjected to burn injury, followed by a single dose of EPO (5000 IU/kg i.p. at day 0 [D0]).
4. Group D (burn + daily EPO dosage, n = 6) rats were subjected to burn injury, followed by a daily dose of EPO for 7 days (3000 IU/kg/day i.p. at D0–D6).

**Fig 1:**
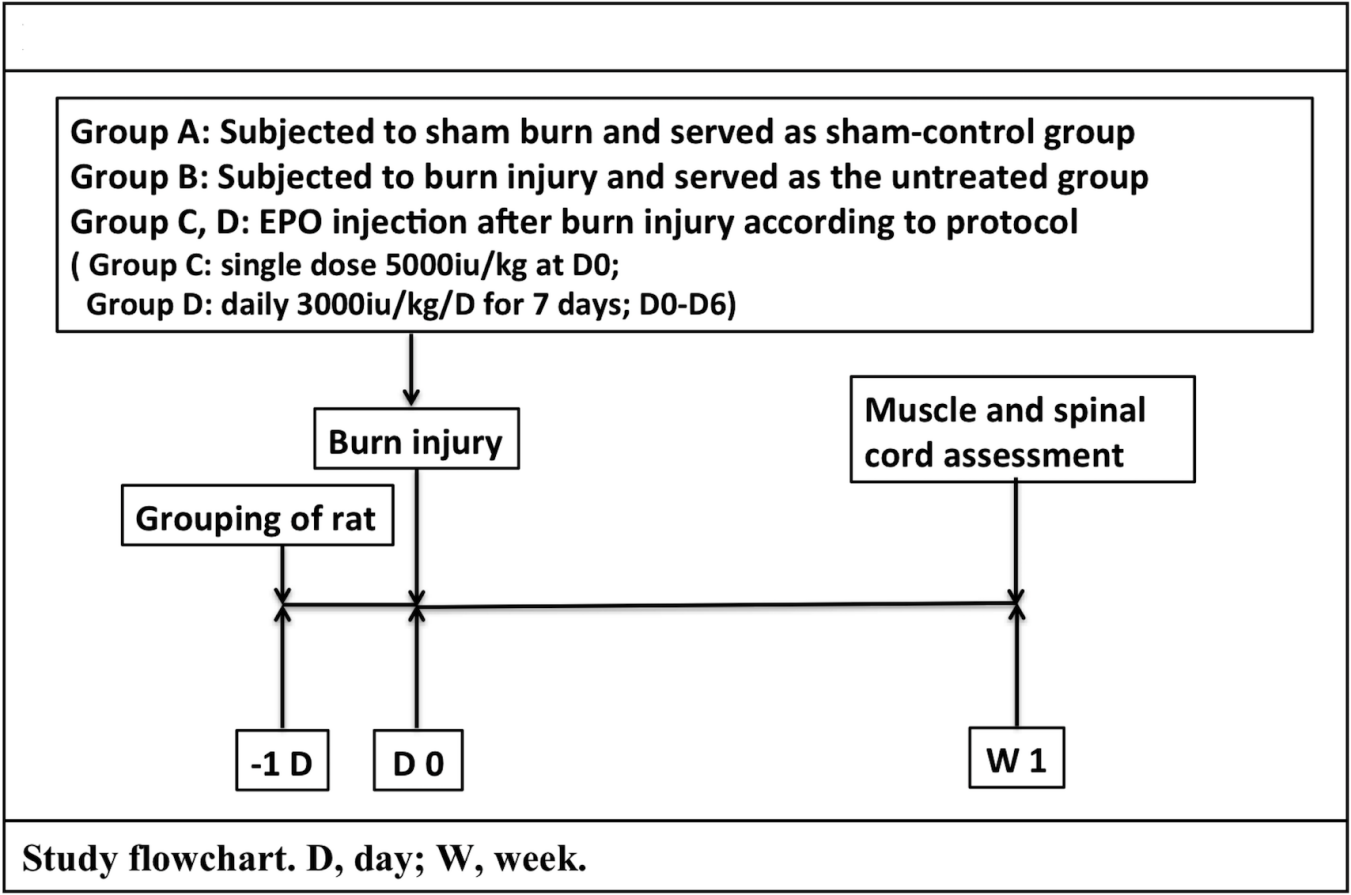
Grouping and flow chart of the animal study procedure. Sham burn or burn injury was induced at day 0. Following the procedure, wound care with 1% SSD was repeated twice a day. EPO was given for pharmacological investigation in Group C and D with different regimens. After completion of the protocol, all rats were sacrificed on week 1 (W1). Spinal cord ventral horn and gastrocnemius muscles were taken and processed by histopathological examination or Western blotting. Abbreviation: D, day; W, week.

Fig 1 shows the basic design of the animal study. An SD rat model of full-thickness burn injury, which induces motor neuron apoptosis in the spinal cord ventral horn, was established according to our previous study [14] Briefly, the rats were subcutaneously anesthetized with Zoletil 50 (50 μg/g; Virbac Laboratory, Carros, France) at day 0. Burn injury was induced by placing the plantar side of the right hind paw on a metal surface with a heated circulating water bath at a temperature of 75 ± 0.5°C, with a 100-g weight placed on the paw to maintain constant contact for 10 s. For Group A, the temperature was changed to 25 ± 0.5°C, and the other steps were same as those for the other three groups. Following the procedure, standard wound care of 1% sliver sulfadiazine cream (Silverdin, Deva, Sliver sulfadizine, 10mg/g) was applied topically. Wound treatment and assessment were repeating twice a day till scarified. All rats returned to their home cages for recovery with free access to chow and tap water. Carprofen (5mg/kg, Rimadyl^®^) was administered for analgesia on the day of burn injury and the next 2 days. On the 7^th^ day (W1), all animals were euthanized by an overdose of Zoletil 50. No animals died during the period.

### EPO

Epoetin (recombinant human EPO, Recormon, Roche) was mixed with 1 mL of 0.9% sodium chloride to achieve the final concentration. The freshly prepared solution was stored at a maximum temperature of 8°C for 45–60 min until further use. EPO was administered intraperitoneally after burn injury.

### Blood samples

Blood samples were collected from the tail vein and analyzed for complete blood count on W1 after burn injury. The samples were analyzed using commercially available clinical assay kits with an autoanalyzer (Bayer ADVIA 2120), the manufacturers’ instructions.

### Western blotting

The rats were sacrificed on W1 by administering an overdose of Zoletil 50. The ventral horn areas of the lumbar 3, 4, and 5 (L3–5) spinal cords and gastrocnemius muscles were separated, frozen in liquid nitrogen, and stored at -80°C. For B-cell lymphoma-2 (BCL-2), BCL-2-associated X protein (BAX), cleaved caspase-3, AKT, p-AKT, mTOR and EPOR assessments, the L3–5 spinal cord ventral horn specimens were homogenized in T-PER Protein Extraction Reagent (Thermo Scientific, Rockford, IL, USA) in the presence of a protease inhibitor and were subsequently incubated. For cleaved caspase-3 assessment in muscle level, the gastrocnemius muscles were prepared as described previously. The samples were centrifuged at 13,000× RPM at 4°C for 30 min. Each protein concentration of the supernatants was measured using bovine serum albumin as the standard. For Western blotting, equal amounts of the total protein content were separated through sodium dodecyl sulfate–polyacrylamide gel electrophoresis (15%) and transferred onto membranes. After being blocked for 1 h with 5% nonfat milk, the membranes were incubated overnight at 4°C with primary antibodies of BAX (1:1000, ProteinTech Group, Chicago, IL, USA), BCL-2 (1:1000, Abcam, Cambridge, MA, USA), cleaved caspase-3 (1:1000, Cell Signaling, Boston, MA, USA), AKT (1:1000, Cell Signaling), p-AKT (1:1000, Cell Signaling), mTOR (1:1000, Cell Signaling), and EPOR (1:1000, Santa Cruz, Santa Cruz, CA, USA). β-actin (1:20,000, Sigma-Aldrich, Saint Louis, MO, USA) was used as an internal control. After washing with Tris-buffered saline with 0.1% Tween-20, secondary antibodies, namely goat antirabbit-horseradish peroxidase (HRP) and goat antimouse-HRP, were applied for 1 h at room temperature. The peroxidase activity was visualized using the ECL Western Blotting Detection kit and Bio-Rad ChemiDoc XRS system. Band intensity was quantified and plotted using Quantity One software, and the average band intensity was obtained from three independent experiments.

### Immunohistochemical staining

The rats were euthanized, and subsequently the ventral horn areas of spinal cord segments (L3–5) were collected on W1 and postfixed overnight in 4% paraformaldehyde in 0.1 M phosphate-buffered saline at 4°C before being transferred into a 30% sucrose solution. To detect microglia activation, the sample sections were double-labeled for phosphorylated p38 mitogen-activated protein kinase (MAPK) (pp38 MAPK, 1:200; Cell Signaling) and oxycocin-42 (OX-42, microglia marker, 1:200; Serotec). To detect the anti-inflammation reaction in neurons, the sample sections were incubated with a mix of iNOS (1:200, Abcam), COX-2 (1:200, Cell Signaling), and monoclonal NeuN (neuron cell marker, 1:1000; Millipore, Temecula, CA, USA). To examine the role of autophagy, antimicrotubule-associated protein light chain-3 (LC3B) rabbit polyclonal antibody (1:200, Cell Signaling), autophagy protein 5 (ATG5, 1:200; Thermo), and monoclonal NeuN (1:1000; Millipore) were used. An appropriate secondary antibody conjugated with goat antirabbit Cy3 (red; Millipore) and goat antimouse Alexa Flour 488 (green, Invitrogen, Carlsbad, CA, USA) was added. Images were acquired using a fluorescence microscope (Leica DM 16000).

### Statistical analysis

Experimental data are expressed as the mean ± standard error of the mean (SEM). SPSS (ver. 14.0, Chicago, IL, USA) was used for the statistical analysis. All data were calculated according to the numerical data, as presented in the text, figures, and figure legends. The bar graphs and errors bars represent the means and standard deviations, respectively. The Western blotting measurements were evaluated using one-way analysis of variance and Tukey pairwise comparison with p < 0.05 considered statistically significant.

## Results

### Single-dose EPO treatment dose not increase hematocrit and red blood cell count

EPO can stimulate red blood cell (RBC) production. A single dose of EPO in Group C did not significantly increase RBC mass compared with Group B. RBC mass increased significantly in Group D compared with Group B. The effect of EPO on hematocrit (Hct) levels was the same as that on RBC mass. Single-dose EPO administration in Group C did not yield significant changes in Hct levels compared with those in Groups A and B. However, Hct levels were significantly higher in Group D than in Group B. Burn injury increased the white blood cell (WBC) count in Group B; however, EPO may prevent a rise in the WBC count. Groups C and D had a significantly lower WBC count than Group B (Table 1).

**Table 1:** Effect of EPO on hematological parameters.

	Group A	Group B	Group C	Group D	*p*^B-A^	*p*^C-B^	*p*^D-B^
**Hct (%)**	40.90±2.40	45.4±2.40	47.70±1.13	57.90±2.79	0.022*	0.252	0.037*
**RBC (x10**^**6**^**/μL)**	7.67±0.23	8.03±0.31	8.15±1.04	9.99±0.69	0.475	0.852	0.041*
**WBC (x10**^**3**^**/μL)**	8.34±0.24	11.49±1.18	7.03±0.59	7.20±2.72	0.037*	0.003**	0.021*
**PLT(x10**^**3**^**/μL)**	798.50±30.41	635.5±74.25	647.50±82.73	714.50±20.43	0.174	0.074	0.174

Data are presented as the mean ± SEM.

p^B-A^: burn-induced (Group B) versus sham-control (Group A) groups, p^C-B^: burn + single-dose EPO group (Group C) versus Group B, and p^D-B^: burn + daily EPO dosage group (Group D) versus Group B. Hct: Hematocrit, RBC: Red blood cell, WBC: White blood cells, PLT: Platelets.

*p < 0.05.

**p < 0.01.

### Effects of EPO on neuroinflammation

Evidence has suggested that excessive neuroinflammation exacerbates neurodegeneration after trauma or in some progressive diseases, such as Alzheimer and Parkinson diseases and glaucoma [44–49]. Therefore, decreased neuroinflammation can ameliorate these disorders.

#### EPO modulates burn-induced microglia activation

**Fig 2:**
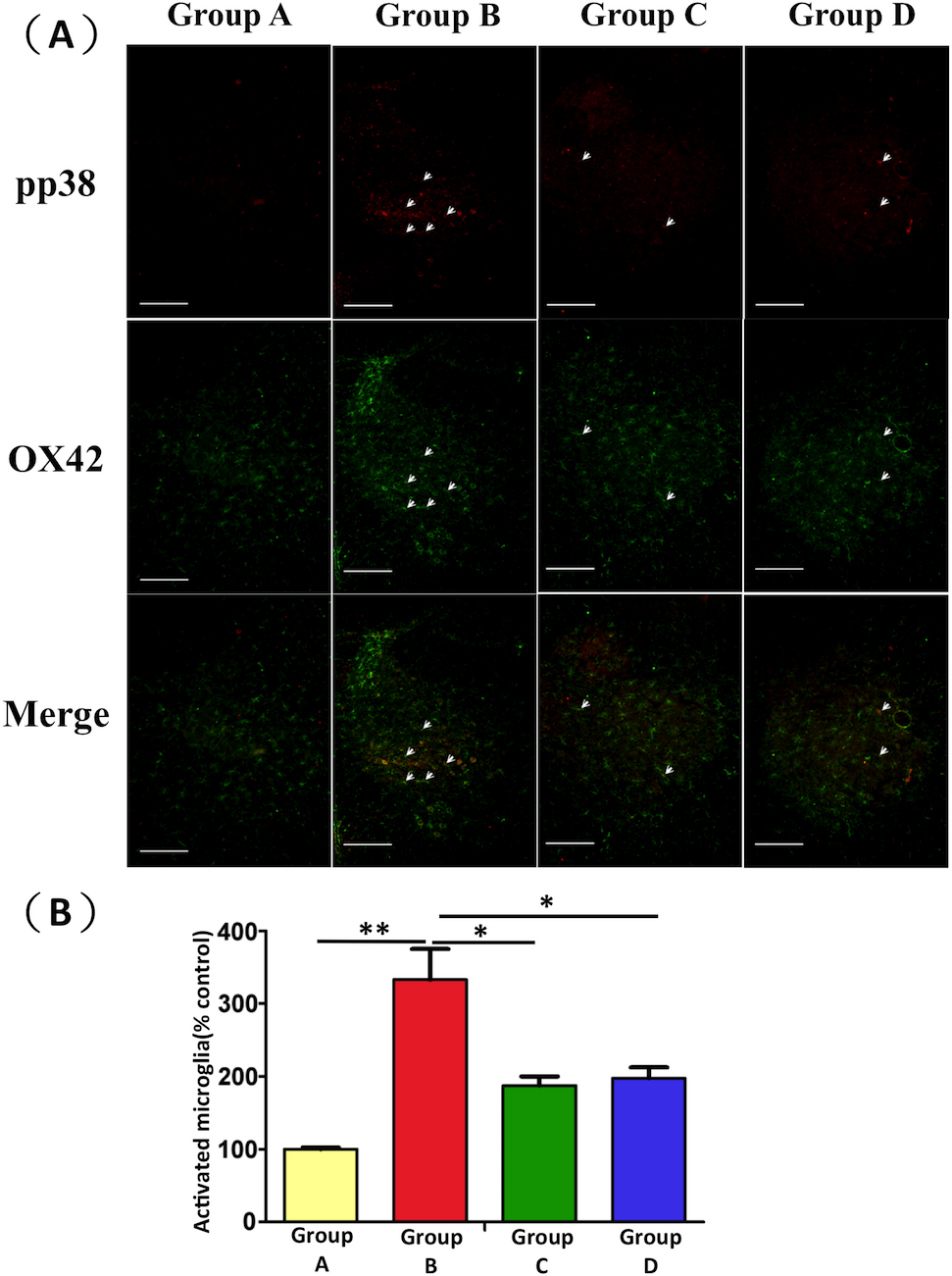
Immunofluorescence staining results for detecting activated microglia expression in the spinal cord ventral horn. (A) Increased expression of activated microglia was observed in Group B compared with Group A. EPO administration resulted in lower microglia expression in Groups C and D compared with Group B (scale bars: 100 μm). (B) Quantitative analysis of activated microglia revealed a significant increase in the number of activated microglia in Group B compared with Group A. EPO treatment attenuated microglia activation. Values are expressed as a percentage of the mean ± SEM (n = 6). *: p < 0.05; **: p < 0.01.

To determine the effects of EPO on microglia activation, p-p38 MAPK and OX-42 expression in the spinal cord ventral horn were analyzed immunohistochemically. The results revealed that p-p38 MAPK was colocalized with OX-42 (Fig 2A). Microglia was significantly activated after burn injury (Group B versus Group A, p < 0.01). Expression of p-p38 MAPK and OX-42 was suppressed in the spinal cord ventral horn of Groups C and D compared with Group B (Group C versus Group B, p < 0.05; Group D versus Group B, p < 0.05; Fig 2B).

#### EPO reduces the expression of iNOS and COX-2 in the spinal cord ventral horn

**Fig 3:**
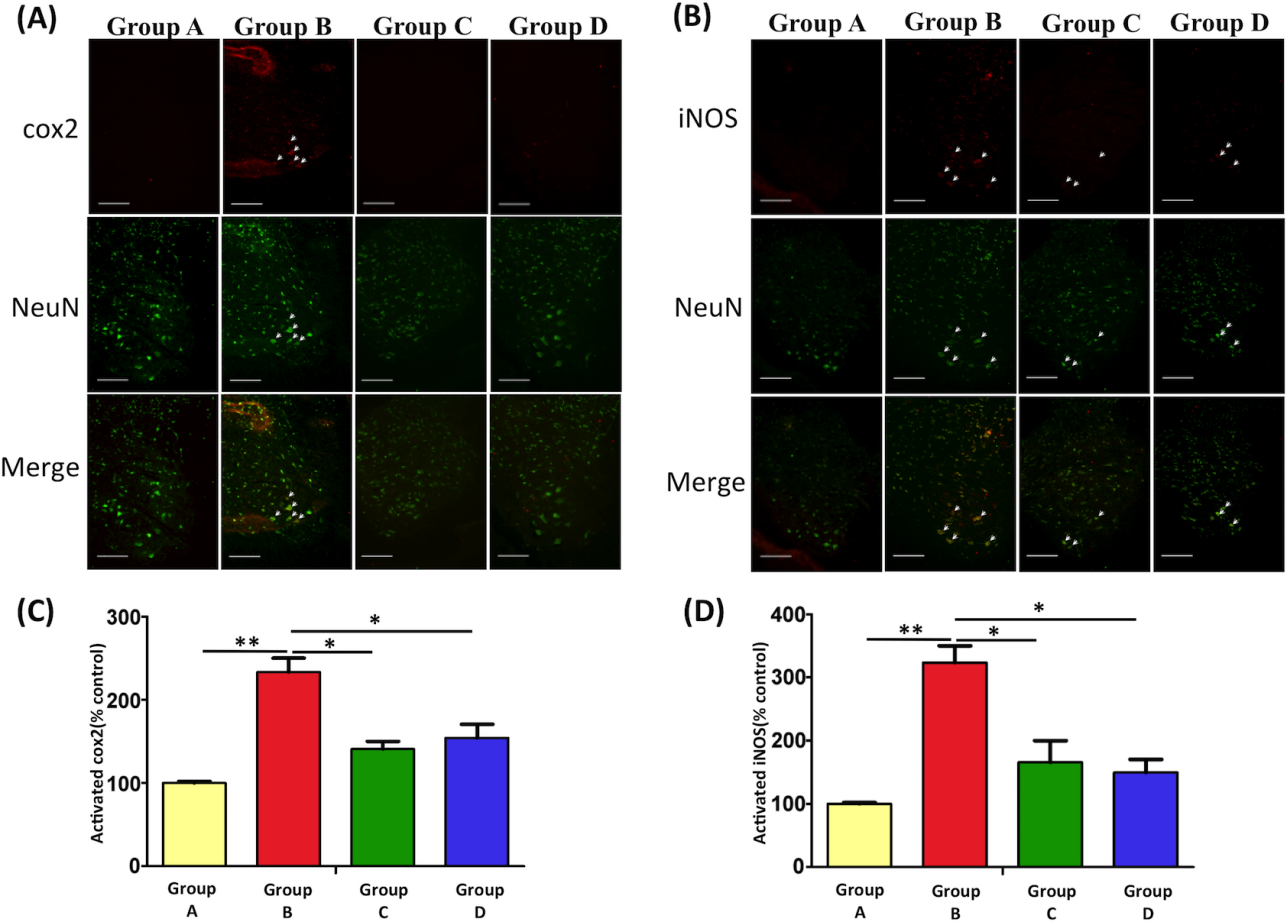
Immunofluorescence staining images and quantitative analysis of COX-2 and iNOS in the spinal cord ventral horn. The immunofluorescence staining of COX-2 (A) and iNOS (B) 7 days after burn injury. The quantitative analysis of COX-2 (C) and iNOS (D) from spinal cord tissues in each experimental group has been assessed. COX-2 and iNOS expression decreased in the EPO-treated groups (Groups C and D) compared with the untreated group (Group B). *: p < 0.05; **: p < 0.01.

As shown in Fig 3, we investigated the effects of EPO on iNOS and COX-2 expression in spinal cord ventral horn (L3–5) through Immunohistochemical staining. Significant increases in iNOS and COX-2 expression were observed after burn injury (Group B versus Group A, p < 0.01). However, after EPO treatment in Groups C and D, COX-2 and iNOS levels decreased significantly (both versus Group B, p < 0.05).

### EPO attenuates burn-induced motor neuron apoptosis

**Fig 4:**
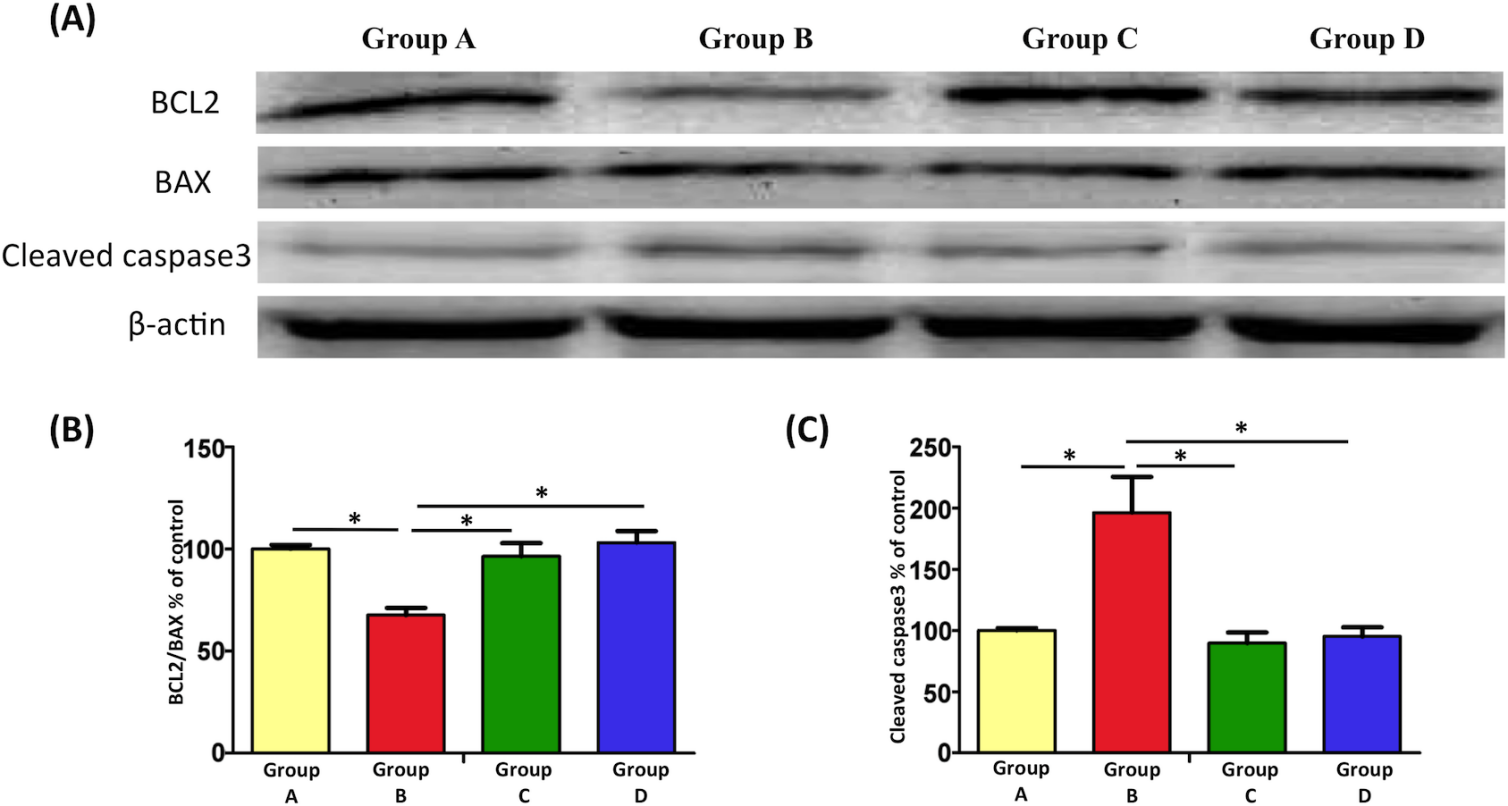
EPO attenuates burn-induced motor neruon apoptosis. (A) Expression levels of BCL-2, BAX, and cleaved caspase-3 in the spinal cord ventral horn, measured through Western blotting. (B) Significant reduction in BCL-2/BAX expression was observed in Group B compared with Group A (100%). However, BCL-2/BAX expression was upregulated after EPO treatment. (C) Cleaved caspase-3 expression increased in the burn-induced group compared with the sham-control group. Similar to the notable reduction in cleaved caspase-3 expression following EPO treatment, a decrease in cleaved caspase-3 was observed. *: p < 0.05.

Our previous study demonstrated burn-induced motor neuron apoptosis through the double immunofluorescence staining of caspases 3 and 9, terminal transferase dUTP nick end labeling (TUNEL) assay, and NeuN staining [14]. In the present study, we investigated the expression levels of BCL-2, BAX, and cleaved caspase-3 in the spinal cord ventral horn after EPO treatment through Western blotting (Fig 4). BCL-2 and BAX, members of the BCL-2 protein family, are anti-apoptotic and pro-apoptotic factors, respectively [50, 51]. Our results indicated that burn injury reduced the BCL-2/BAX ratio (Group B versus Group A, p < 0.05), and EPO treatment reversed this phenomenon significantly (Groups C and D versus Group B, both p < 0.05). In addition, EPO reduced the expression of cleaved caspase-3 in Groups C and D compared with Group B (both p < 0.05), indicating that EPO reduced cleaved capase-3 expression by upregulating BCL-2 expression and downregulating BAX expression.

### EPO reduces burn injury-induced autophagy in the spinal cord ventral horn

**Fig 5:**
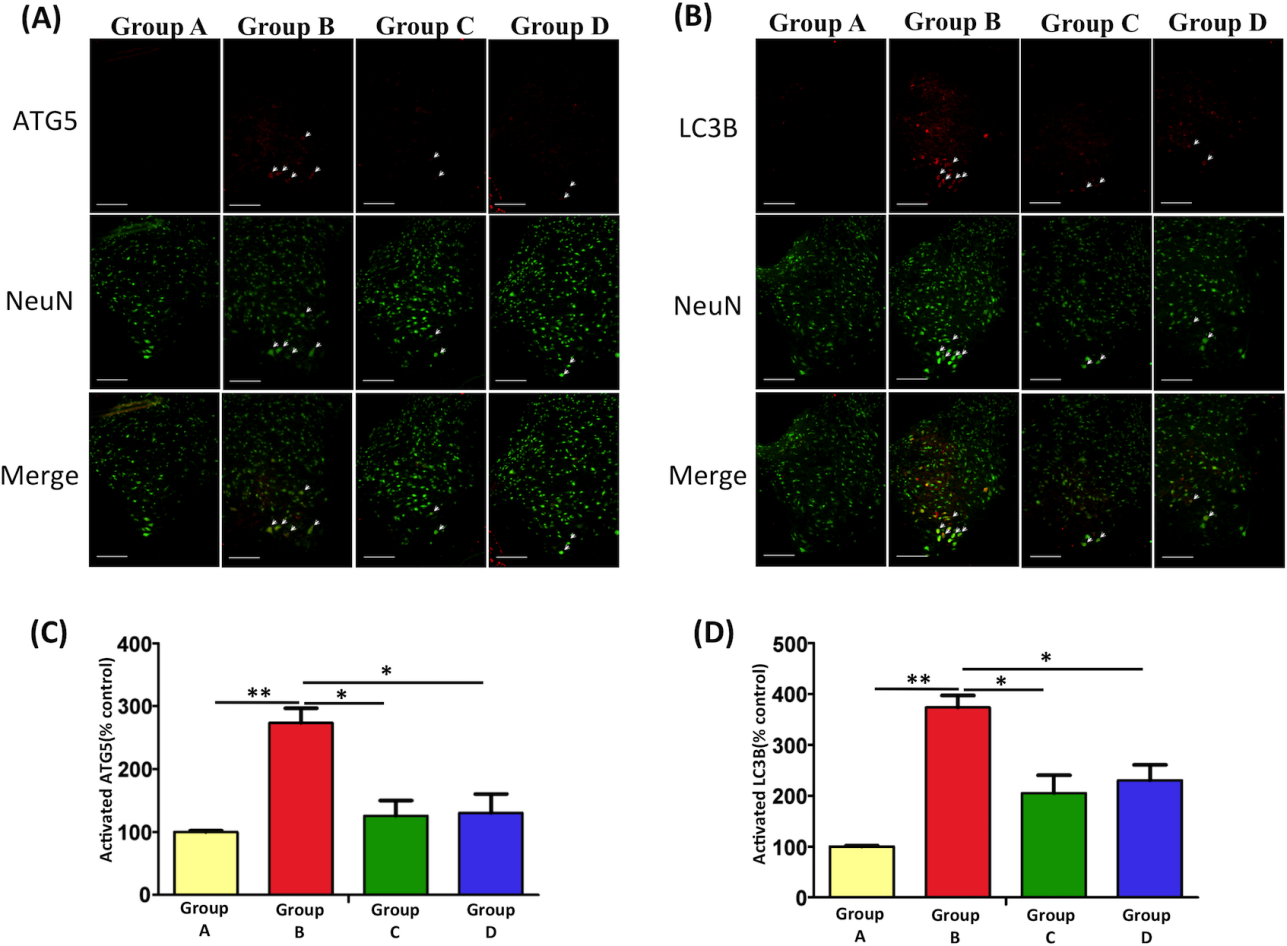
The Effects of EPO on autophagy markers in burn injury model by immunofluorescence analysis. (A, B) Double immunofluorescence staining and merged images using LC3B, ATG5, and NeuN in the spinal cord ventral horn were shown. The quantitative analysis of ATG5 (C) and LC3B activity (D) were measured. Burn injury significantly increased LC3B and ATG5 immunoreactivity in Group B versus Group A. After EPO treatment, LC3B and ATG5 immunoreactivity decreased markedly in Groups C and D vs Group B (**: p < 0.01; *: p < 0.05 versus Group B; Group A: 100%). Scale bars: 50 μm.

Autophagy plays a crucial role in the regulation of cell death pathways and neurodegeneration. We used a burn injury-induced motor neuron apoptosis model to investigate whether the autophagy pathway is involved. The results demonstrated that burn injury can significantly induce an increase in autophagy, and neurons were costained with autophagic biomarkers, including ATG5 and LC3B, in the spinal cord ventral horn (p < 0.01 versus Group A, Fig 5). In addition, our data suggest that the downregulation in autophagy after EPO treatment may be a neuroprotective mechanism mediated through the inhibition of programmed cell death (Group C versus Group B, p < 0.05; Group D versus Group B, p < 0.05).

### EPO modulates the AKT-mTOR pathway to suppress programmed cell death in the spinal cord ventral horn

**Fig 6:**
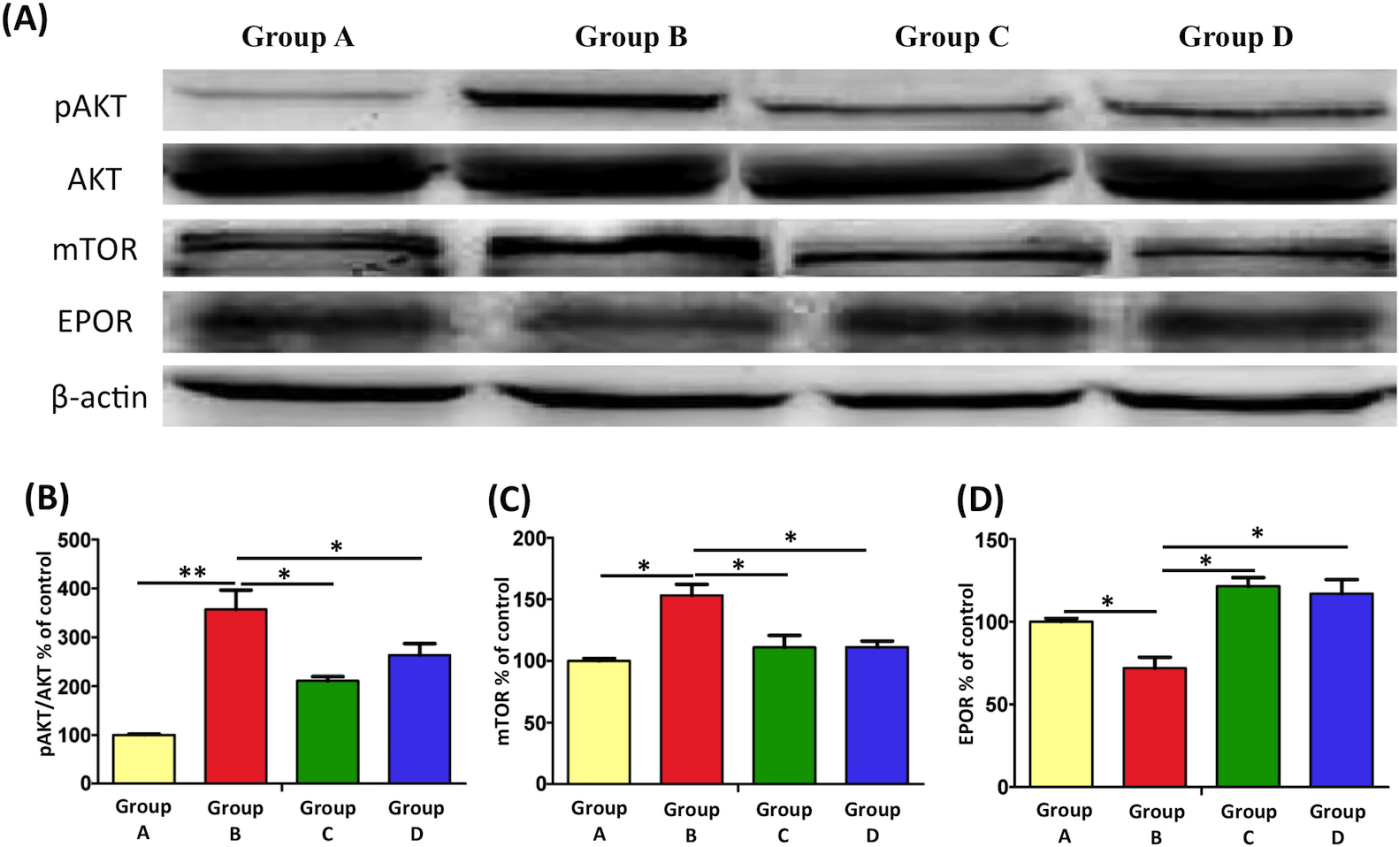
Western blotting and relative expression levels of pAKT/AKT, mTOR, and EPOR. (A) Representative result from Western blotting in the spinal cord ventral horn after burn injury and EPO administration. (B, C) The activity of p-AKT/AKT and mTOR by Western blotting. It revealed a remarkable increase in p-AKT/AKT and mTOR expression after burn injury. However, this burn-induced increased expression was blocked after EPO treatment. (D) The effect of EPO on EPOR activation in spinal cord. It showed the expression of EPOR increased after EPO treatment (*p < 0.05; **p < 0.01; Group A: 100%).

The AKT-mTOR pathway is a critical pathway in the regulation of cell survival. AKT is involved in the inhibition of neuronal death, and mTOR is a downsteam effector of AKT that controls protein systhesis. Previous findings have suggested that AKT and mTOR might regulate programmed cell death [52, 53]. We therefore assessed their role in motor neuron death after burn injury. In Group B, burn injury significantly increased the immunoreactivity of p-AKT/AKT and mTOR (Fig 6A–6C; **: p < 0.01 and *: p < 0.05).

We further examined the effects of EPO on mTOR activity and its upstream regulator signaling kinase, p-AKT in the burn injury model (Fig 6A–6C). The results revealed that increasement of p-AKT/AKT and mTOR expression following burn injury was abolished after EPO treatment (p < 0.05), suggesting that pharmacologically high EPO concentrations modulate the AKT-mTOR pathway to provide EPO-induced cytoprotection.

EPO exerts its cytoprotective effects by interacting with specific EPORs, which belong to the single-transmembrane cytokine receptor family [54]. EPORs have been identified on various cell types, including renal and endothelial cells, cardiomyocytes, neurons, astrocytes, and microglia [55–58]. In the study, EPO administration increased EPOR expression (Fig 6A and 6D) and AKT-mTOR signaling molecules might involve the downstream signaling with EPO treatment. However, more evidences will be needed to elucidate the direct EPO-EPOR interaction for neuroprotection in the model.

### Effect of EPO on burn-induced muscle cells apoptosis

**Fig 7:**
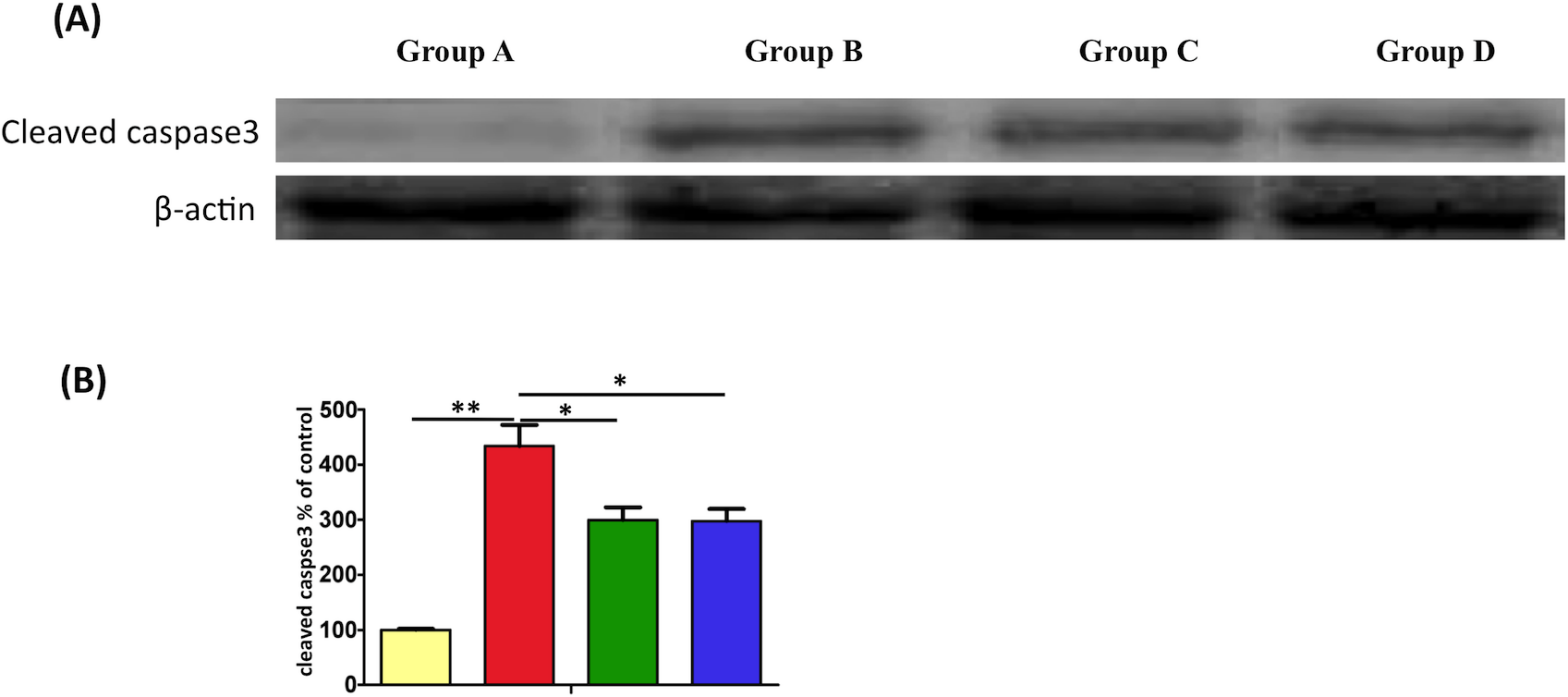
EPO alleviated burn-induced muscle cells apoptosis by Western blotting. Burn injury significantly increased the expression of cleaved caspase-3 in muscle cells vs Group A (p < 0.01), and Groups C and D exhibited decreased cleaved caspase-3 expression (p < 0.05) vs Group B.

Burn injury significantly increased the expression of cleaved caspase-3 in muscle cells (p < 0.01), and Groups C and D exhibited decreased cleaved caspase-3 expression (p < 0.05; Fig 7).

## Discussion

The exact mechanisms through which EPO modulates muscle wastage and motor neuron apoptosis in the spinal cord ventral horn after burn injury are not yet completely understood. Our present work suggests EPO inhibits programmed cell death, including apoptosis and autophagy. In addition, EPO exerts neuroprotective effects in the burn injury-induced motor neuron damage model through several other mechanisms, such as the modulation of microglia activation, iNOS/COX-2 synthesis, and the AKT-mTOR pathway. It also alleviated muscle cell apoptosis post-burn.

The optimal EPO dose for *in-vivo* tissue protection is yet to be determined. Previous studies have reported that a relatively high EPO dose is required to promote maximal anti-apoptotic effects. A single high dose (3000 IU/kg) of EPO may provide neuroprotection, whereas lower EPO doses are insufficient for alleviating cerebral white matter inflammation [55]. Multiple doses of EPO may improve neuroimage findings and motor function in neonates with hypoxic-ischemic encephalopathy [56, 57]. Studies have reported that EPO administration (5000 IU/kg) after injury attenuates early-phase tissue damage [58–60] and improves the long-term neurological outcomes in acute ischemic stroke [61]. A single dose (5000 IU/kg single-dose regimen; Group C) or multiple doses regimen of EPO (3000 IU/kg 7-day regimen; Group D) was adopted in present study to survey the efficacy and safety of adjunctive EPO. Our short-term outcomes at 7 day post-burn suggest that treatments with two different doses of EPO could attenuate programmed cell death.

To our knowledge, only one study compared two dose regimens (5000 IU/kg, single dose versus daily doses for 3 days) of EPO were injected at 1 day after traumatic brain injury and showed that 3-days regimen provided better functional recovery and preservation of hippocampal neuron than a single-dose regimen [62]. In our present work, the neuroprotective efficacy in Group D of daily EPO injection was similar in Group C of single-dose EPO at day 7 post-burn. One possible reason is the protective effect in Group D could persist longer than in Group C. The beneficial neurocognitive effects of repeated EPO treatment were demonstrated and produced consistent and long-lasting improvement functional outcomes [63–65]. In addition, although EPO has a known dose response for neuroprotection, the possibility of the effect is a non-linear dose-response relationship in higher repeat dose regimen. Previous retrospective study showed that extremely low birth weight infants received EPO (250 to 400 IU/kg×3/week ×6 weeks) and their development index correlate with cumulative EPO exposure [63]. However, the median 6-week cumulative EPO dose was 3750 IU/kg. In traumatic brain injury model, EPO with a range of 1000 to 7000 IU/kg was used and the medium dose of EPO (5000 IU/kg) showed a significant improvement in histological and functional outcomes compared with the lower or higher EPO dose groups [66]. Owing to complex pharmacokinetic/pharmacodynamics behavior of EPO [67] and limited capacity for EPO-tis receptor binding at higher EPO concentrations. We speculate there is saturation of the receptors in the EPO binding and resulting diminished efficacy in Group D with daily repeated high dose EPO exposure.

Systemic repeated EPO treatment may be associated with some side effects such as polycythemia resulting from its erythropoietic activity. In some cases, polycythemia could lead to thrombotic complications and high blood pressure. We investigated the possible adverse effects of systemic single or daily EPO treatment on hematological parameters. Leukocyte count was decreased under EPO treatment in Group C and D (p<0.05 versus Group B). In patients with chronic renal disease, decreased low-grade inflammation was reflected by reduced WBC counts following EPO treatment [68]. However, polycythemia and elevated hematocrit level were noted in Group D (*p*<0.05 versus Group B), which increases blood viscosity and the probability of thromboembolic events. Systemic single dose EPO (5000 IU/kg) injection may be effective as well as safe in the model.

Our previous study has demonstrated burn-induced motor neuron apoptosis up to 8 weeks after injury by using TUNEL assay [14]. In the present study, we further examined the expression of pro-apoptotic (BAX) and anti-apoptotic (BCL-2) proteins, which are a part of the intrinsic apoptotic pathway, and cleaved caspase-3, which is considered a hallmark of apoptosis. The neuroprotective effects of EPO observed in Groups C and D were partly associated with anti-apoptosis.

Cellular stress-induced autophagy is a fundamental catabolic process in cellular organelle homeostasis; however, its underlying mechanism is yet to be determined. Autophagy can be activated by starvation and a variety of stress to remove macromolecular damage. Inadequate (both insufficient and excessive) autophagy expression has been associated with diseases. Previous studies have observed that the expression of autophagy signals increases significantly in mouse liver and heart models after severe burn injury [69, 70]. Abnormal autophagy expression has been observed in critically ill rabbits after thermal injury [71]. In addition, skeletal biology studies have demonstrated dysregulated autophagy play a role in osteoarthritis and an acute increase in autophagy may be responsible for compensatory responses to cellular stress. However, the suppressed activation of autophagy during prolonged stress exceeds the capacity of the mechanism and may lead to further degeneration [72, 73]. Therefore, autophagy regulation is a potential therapeutic target. Our short-term results showed that burn injury induces the expression of autophagic markers, LC3B and ATG5 in the spinal cord ventral horn, and their expression is reduced with EPO treatment.

The process of inflammation onset following burn injury is also involved in pathogenesis. The severity of the CNS insult correlates strongly with the robustness of microglia activation and proinflammatory cytokine production. In acute or chronic neuroinflammation, microglia act as important mediators. The over-activation of microglia exacerbates inflammatory effects and mediates cell degeneration, leading to neuron death [74–76]. In our study, burn injury increased microglia activation and may have partly contributed to neuronal damage. Moreover, the pharmacological down-regulation of microglia activation can be a potential therapeutic regimen. In some neuromuscular diseases, neuroinflammation plays an important role in pathogenesis. The inhibition of microglia activation leads to the down-regulation of proinflammatory markers and delayed neuron death [77–80]. A recent study reported that EPO attenuates microglia activation through morphological changes, phagocytosis, and inflammatory cytokine production [81]. Our data showed that EPO-treated groups exhibited a significant improvement in microglia activation and neuronal death compared with burn injury groups. On the basis of the present findings, we propose that the pleiotropic function of EPO may be involved in protecting against neuroinflammation.

iNOS is a major downstream mediator of inflammation after major trauma, including burn injury. A iNOS-knockout mice study showed that iNOS may contribute to the burn-induced development of inflammatory response and apoptotic changes in skeletal muscles [13]. The present results reveal that iNOS expression increases in the spinal cord ventral horn after burn injury, and EPO treatment results in a decrease in iNOS levels. Furthermore, COX-2 is induced on pathogenic stimulation and participates in the synthesis of prostaglandins, which are associated with proinflammatory activities. Increased COX-2 expression has been suggested to be involved in the neurodegenerative processes of several acute and chronic diseases. According to the IHC findings of the present study, COX-2 expression increases after burn injury, demonstrating its crucial role in aggravating inflammatory responses. EPO-treated groups decreased COX-2 expression. In conclusion, our current data indicate that EPO treatment exerts anti-inflammatory capacity. We propose that burn injury induces iNOS and COX-2 expression, and EPO treatment can ameliorate the inflammation through two mechanisms: by down-regulating iNOS synthesis and by controlling COX-2 production, which regulates proinflammatory cytokine cascade activation.

The molecular pathways initiated by EPO for neuroprotection are still under active investigation. In chronic constriction injury model, Schwann cells express EPOR and represent a major target for exogenous EPO [82]. EPO activates the EPOR to induce cellular signaling and block apoptosis [21, 83]. An increase EPOR phosphorylation by exogenous EPO administration maintains cell survival in ischemic hippocampi of rats [84]. However, some data suggest that EPO may act on additional receptors or independently on receptor to trigger multiple intracellular signal cascades [80]. In present study, we showed elevated expression of EPOR after EPO injection, while more evidences will be needed to proof the direct role of EPOR in neuroprotection. Furthermore, the phosphoinositide 3-kinase-AKT-mTOR pathway plays a crucial role in regulating cellular growth, differentiation, adhesion, and inflammatory reactions [85–87]. In a traumatic brain injury model, AKT and mTOR were activated after injury [53] and inhibition of AKT and mTOR improved motor and cognitive deficits post-injury. Similar in our results revealed that p-AKT/AKT and mTOR levels significantly increased in the spinal cord ventral horn after burn injury. By contrast, EPO treatment reduced AKT-mTOR signaling in the burn injury model.

There are abundant data indicated that EPO have neuroprotective activities after traumatic brain injury, stroke, and neurodegenerative diseases. Scanty experimental evidences and clinical studies indicated its neuroprotective effects after thermal injury. Further researches will be performed in the future to solve following questions. First, the optimal therapeutic dose and the appropriate timing of EPO were not determined to reach maximal protective effect and not to create drug saturation. Second, the role of EPOR in participating EPO’s neuroprotection remains to be elucidated. Third, we survey short-term effect of EPO treatment on motor neuron apoptosis after thermal injury. Our data suggest systemic single or daily EPO application was sufficient to improve spinal cord ventral horn motor neuron inflammation and cell death at 7 days follow-up. However, a significant increase of hematocrit was noted in systemic daily treated group. Previous data suggested that locally EPO application is able to suppress cell death without increasing side effects [88]. Further studies will be needed to compare the efficacy and safety between systemic and local EPO treatment.

## Conclusion

The present study reveals that EPO modulates inflammation by reducing iNOS and COX2 levels, inhibiting microglia activation, and suppressing autophagy activation. In addition, we propose that the EPO-mediated alleviation of motor neuron apoptosis may involve the AKT-mTOR pathway. EPO is a pleiotropic protein that can influence programmed cell death, glial reactivity, and iNOS or COX2 levels simultaneously.
